# Harnessing heterologous and endogenous CRISPR-Cas machineries for efficient markerless genome editing in *Clostridium*

**DOI:** 10.1038/srep25666

**Published:** 2016-05-09

**Authors:** Michael E. Pyne, Mark R. Bruder, Murray Moo-Young, Duane A. Chung, C. Perry Chou

**Affiliations:** 1 Department of Chemical Engineering, University of Waterloo, Waterloo, Ontario, Canada; 2 Department of Pathology and Molecular Medicine, McMaster University, Ontario, Canada; 3 Algaeneers Inc. and Neemo Inc., Hamilton, Ontario, Canada

## Abstract

Application of CRISPR-Cas9 systems has revolutionized genome editing across all domains of life. Here we report implementation of the heterologous Type II CRISPR-Cas9 system in *Clostridium pasteurianum* for markerless genome editing. Since 74% of species harbor CRISPR-Cas loci in *Clostridium*, we also explored the prospect of co-opting host-encoded CRISPR-Cas machinery for genome editing. Motivation for this work was bolstered from the observation that plasmids expressing heterologous *cas9* result in poor transformation of *Clostridium*. To address this barrier and establish proof-of-concept, we focus on characterization and exploitation of the *C. pasteurianum* Type I-B CRISPR-Cas system. *In silico* spacer analysis and *in vivo* interference assays revealed three protospacer adjacent motif (PAM) sequences required for site-specific nucleolytic attack. Introduction of a synthetic CRISPR array and *cpaAIR* gene deletion template yielded an editing efficiency of 100%. In contrast, the heterologous Type II CRISPR-Cas9 system generated only 25% of the total yield of edited cells, suggesting that native machinery provides a superior foundation for genome editing by precluding expression of *cas9 in trans*. To broaden our approach, we also identified putative PAM sequences in three key species of *Clostridium*. This is the first report of genome editing through harnessing native CRISPR-Cas machinery in *Clostridium*.

Clustered Regularly Interspaced Short Palindromic Repeats (CRISPRs) and CRISPR-associated (Cas) proteins comprise the basis of adaptive immunity in bacteria and archaea^1^,^2^. CRISPR-Cas systems are currently grouped into six broad types, designated Type I through VI^3^,^4^. CRISPR-Cas Types I, II, and III, the most prevalent systems in both archaea and bacteria^3^, are differentiated by the presence of *cas3*, *cas9*, or *cas10* signature genes, respectively^5^. Based on the composition and arrangement of *cas* gene operons, CRISPR-Cas systems are further divided into 16 distinct subtypes^3^. Type I systems, comprised of six distinct subtypes (I-A to I-F), exhibit the greatest diversity^6^ and subtype I-B is the most abundant CRISPR-Cas system represented in nature^3^. CRISPR-Cas loci have been identified in 45% of bacteria and 84% of archaea^7^ due to widespread horizontal transfer of CRISPR-Cas loci within the prokaryotes^8^.

CRISPR-based immunity encompasses three distinct processes, termed adaptation, expression, and interference^9^,^10^. Adaptation involves the acquisition of specific nucleotide sequence tags, referred to as protospacers in their native context within invading genetic elements, particularly bacteriophages (phages) and plasmids^11^,^12^,^13^. During periods of predation, protospacers are rapidly acquired and incorporated into the host genome, where they are subsequently referred to as spacers^14^. Cas1 and Cas2, which form a complex that mediates acquisition of new spacers^15^, are the only proteins conserved between all CRISPR-Cas subtypes^5^. Chromosomally-encoded spacers are flanked by 24–48 bp partially-palindromic direct repeat sequences^6^, iterations of which constitute CRISPR arrays. Up to 587 spacers have been identified within a single CRISPR array^16^, exemplifying the exceptional level of attack experienced by many microorganisms in nature. During the expression phase of CRISPR immunity, acquired spacer sequences are expressed and, in conjunction with Cas proteins, provide resistance against invading genetic elements. CRISPR arrays are first transcribed into a single precursor CRISPR RNA (pre-crRNA), which is cleaved into individual repeat-spacer-repeat units by Cas6 (Type I and III systems)^17^ or the ubiquitous RNase III enzyme and a small trans-activating crRNA (tracrRNA) (Type II systems)^18^, yielding mature crRNAs (Fig. 1). Once processed, crRNAs enlist and form complexes with specific Cas proteins, including the endonucleases responsible for attack of invading nucleic acids during the interference stage of CRISPR immunity. In Type I systems, crRNAs complex with ‘Cascade’ (a multiprotein Cas complex for antiviral defence) and base pair with invader DNA^19^, triggering nucleolytic attack by Cas3^20^. In many CRISPR-Cas subtypes, Cascade includes Cas5, Cas6, Cas7, and Cas8^6^. Type II systems are markedly simpler and more compact than Type I machinery, as the Cas9 endonuclease, tracrRNA, and crRNA, as well as the ubiquitous RNase III enzyme, are the sole determinants required for interference (Fig. 1). Alternatively, crRNA and tracrRNAs can be fused into a single guide RNA (gRNA)^21^. While Cas9 attack results in a blunt double-stranded DNA break (DB)^22^, Cas3 cleaves only one strand of invading DNA, generating a DNA nick (DN). Nicked target DNA is subsequently unwound and progressively degraded by Cas3^23^. Because host-encoded spacer and invader protospacer sequences are often identical, cells harboring Type I and II CRISPR-Cas systems evade self-attack through recognition of a requisite sequence located directly adjacent to invading protospacers, termed the protospacer-adjacent motif (PAM)^24^,^25^. In many organisms, the PAM element is highly promiscuous, affording flexibility in recognition of invading protospacers, whereby specific non-degenerate sequences that constitute the consensus are referred to as PAM sequences. The location of the PAM differs between Type I and II CRISPR-Cas systems, occurring immediately upstream of the protospacer in Type I (i.e. 5′-PAM-protospacer-3′) and immediately downstream of the protospacer in Type II systems (i.e. 5′-protospacer-PAM-3′)^14^,^25^,^26^ (Fig. 1). The site of nucleolytic attack also differs between CRISPR-Cas Types, as Cas9 cleaves DNA three nucleotides upstream of the PAM element^21^,^22^, while Cas3 nicks the PAM-complementary strand outside of the area of interaction with crRNA^20^.

Owing to the simplicity of CRISPR-Cas9 interference in Type II systems, the *S. pyogenes* CRISPR-Cas9 machinery has recently been implemented for extensive genome editing in a wide range of organisms, such as *E. coli*^27^,^28^,^29^, yeast^30^,^31^, mice^32^, zebrafish^33^, plants^34^, and human cells^35^,^36^. In bacteria, CRISPR-based methods of genome editing signify a critical divergence from traditional techniques of genetic manipulation involving the use of chromosomally-encoded antibiotic resistance markers, which must be excised and recycled following each successive round of integration^37^. Within *Clostridium*, a genus with immense importance to medical and industrial biotechnology^38^,^39^, as well as human disease^40^, genetic engineering technologies are notoriously immature, as the genus suffers from overall low transformation efficiencies and poor homologous recombination^41^. Existing clostridial genome engineering methods, based on mobile group II introns, antibiotic resistance determinants, and counter-selectable markers, are laborious, technically challenging, and often ineffective^42^,^43^,^44^. In contrast, CRISPR-based methodologies provide a powerful means of selecting rare recombination events, even in strains suffering from poor homologous recombination. Such strategies have been shown to be highly robust, frequently generating editing efficiencies up to 100%^27^,^29^,^45^. Accordingly, the *S. pyogenes* Type II CRISPR-Cas system has recently been adapted for use in *C. beijerinckii*^46^ and *C. cellulolyticum*^47^, facilitating highly precise genetic modification of clostridial genomes and paving the way for robust genome editing in industrial and pathogenic clostridia.

Here we report development of broadly applicable strategies of markerless genome editing based on exploitation of both heterologous (Type II) and endogenous (Type I) bacterial CRISPR-Cas systems in *C. pasteurianum*, an organism possessing substantial biotechnological potential for conversion of waste glycerol to butanol as a prospective biofuel^48^. While various tools for genetic manipulation of *C. pasteurianum* are under active development recently^49^,^50^, effective site-specific genome editing for this organism is lacking. In this study, we demonstrate the first implementation of *S. pyogenes* Type II CRISPR-Cas9 machinery for markerless and site-specific genome editing in *C. pasteurianum*. Recently, we sequenced the *C. pasteurianum* genome^51^ and identified a central Type I-B CRISPR-Cas locus, which we exploit here as a chassis for genome editing based on earlier successes harnessing endogenous CRISPR-Cas loci in other bacteria^52^,^53^. Our strategy encompasses plasmid-borne expression of a synthetic Type I-B CRISPR array that can be site-specifically programmed to any gene within the organism’s genome. Providing an editing template designed to delete the chromosomal protospacer and adjacent PAM yields an editing efficiency of 100% based on screening of 10 representative colonies. To our knowledge, the approach described here is the first report of genome editing in *Clostridium* by co-opting native CRISPR-Cas machinery. Importantly, our strategy is broadly applicable to any bacterium or archaeon that encodes a functional CRISPR-Cas locus and appears to yield more edited cells compared to the commonly employed heterologous Type II CRISPR-Cas9 system.

## Results

### Implementation of the Type II CRISPR-Cas9 system for genome editing in *C. pasteurianum*

Recently, two groups reported a CRISPR-based methodology employing the Type II system from *S. pyogenes* for use in genome editing of *C. beijerinckii* and *C. cellulolyticum*^46^,^47^. This system requires expression of the *cas9* endonuclease gene *in trans*, in addition to a chimeric guide RNA (gRNA) containing a programmable RNA spacer. To determine if the *S. pyogenes* machinery could also function for genome editing in *C. pasteurianum*, we constructed a Type II CRISPR-Cas9 vector by placing *cas9* under constitutive control of the *C. pasteurianum* thiolase (*thl*) gene promoter and designing a synthetic gRNA expressed from the *C. beijerinckii* sCbei_5830 small RNA promoter^46^. We selected the *cpaAIR* gene as a target double-stranded DB site through the use of a 20 nt spacer located within the *cpaAIR* coding sequence, as this gene has been previously disrupted in *C. pasteurianum*^50^. An *S. pyogenes* Type II PAM sequence (5′-NGG-3′), required for recognition and subsequent cleavage by Cas9^27^, is located at the 3′ end of the *cpaAIR* protospacer sequence within the genome of *C. pasteurianum* (Fig. 2a). Transformation of *C. pasteurianum* with the resulting vector, designated pCas9gRNA-cpaAIR, yielded an average transformation efficiency of 0.03 colony-forming units (CFU) μg^−1^ DNA (Fig. 2b). Only one out of five attempts at transfer of pCas9gRNA-cpaAIR produced a single transformant, indicating efficient Cas9-mediated killing of host cells. To demonstrate genome editing using this system, we constructed pCas9gRNA-delcpaAIR through introduction of a *cpaAIR* gene deletion editing cassette into plasmid pCas9gRNA-cpaAIR. The editing cassette was designed to contain 1,029 bp and 1,057 bp homology regions to the *cpaAIR* locus, which together flank the putative *cpaAIR* double-stranded DB site. Homologous recombination between the plasmid-borne editing cassette and the *C. pasteurianum* chromosome is expected to result in a *cpaAIR* gene deletion comprising 567 bp of the *cpaAIR* coding sequence, including the protospacer and associated PAM element required for Cas9 attack, and 19 bp of the upstream *cpaAIR* gene region, including the putative *cpaAIR* gene promoter (Fig. 2a). Compared to the lethal pCas9gRNA-cpaAIR vector, introduction of pCas9gRNA-delcpaAIR established transformation. A transformation efficiency of 2.6 CFU μg^−1^ DNA was obtained using pCas9gRNA-delcpaAIR, an 87-fold increase compared to pCas9gRNA-cpaAIR (Fig. 2b). Genotyping of 10 pCas9gRNA-delcpaAIR transformants generated the expected PCR product corresponding to *cpaAIR* gene deletion, resulting in an editing efficiency of 100% (Fig. 2c). Sanger sequencing of a single pCas9gRNA-delcpaAIR transformant confirmed successful deletion of a 762 bp region of the *cpaAIR* coding sequence (data not shown).

Despite an editing efficiency of 100% using heterologous Type II CRISPR-Cas9 machinery, an average of only 47 total CFU were obtained by introducing 15–25 μg of pCas9gRNA-delcpaAIR plasmid DNA (2.6 CFU μg^−1^ DNA). Such a low transformation efficiency may impede more ambitious genome editing strategies, such as integration of large DNA constructs and multiplexed editing. Since expression of the Cas9 endonuclease has been shown to be moderately toxic in a multitude of organisms [e.g. mycobacteria, yeast, algae, and mice^32^,^54^,^55^,^56^], even in the absence of a targeting gRNA, we prepared various *cas9*-expressing plasmid constructs to determine if expression of *cas9* leads to reduced levels of transformation. Introduction of a *cas9* expression cassette lacking a gRNA into plasmid pMTL85141 (transformation efficiency of 6.3 × 10^3^ CFU μg^−1^ DNA), generating p85Cas9, resulted in a reduction in transformation efficiency of more than two orders of magnitude (26 CFU CFU μg^−1^ DNA) (Fig. 2b). Modifying the pIM13 replication module of p85Cas9 to one based on pCB102^57^ in plasmid p83Cas9 further reduced transformation to barely detectable levels (0.7 CFU μg^−1^ DNA). Importantly, transformation of *C. pasteurianum* with p85delCas9, constructed through deletion of the putative *cas9* gene promoter in p85Cas9, restored transformation to typical levels (2.2 × 10^3^ CFU μg^−1^ DNA). Collectively these data demonstrate that expression of Cas9 in the absence of a gRNA significantly reduces transformation of *C. pasteurianum*. It is noteworthy that we also observed a dramatically reduced level of transformation of *Clostridium acetobutylicum* using plasmid p85Cas9, which could also be rescued through deletion of the *cas9* gene promoter in p85delCas9 (data not shown).

### Analysis of the *C. pasteurianum* Type I-B CRISPR-Cas system and identification of putative protospacer matches to host-specified spacers

Due to the inhibitory effect of *cas9* expression on transformation, we reasoned that the *S. pyogenes* Type II CRISPR-Cas9 system imposes significant limitations on genome editing in *Clostridium*, as the clostridia are transformed at substantially lower levels compared to most bacteria^41^. To evade poor transformation of *cas9*-encoded plasmids, we investigated the prospect of genome editing using endogenous CRISPR-Cas machinery. We recently sequenced the genome of *C. pasteurianum* and unveiled a CRISPR-Cas system comprised of a 37-spacer CRISPR array upstream of a core *cas* gene operon (*cas6*-*cas8b*-*cas7*-*cas5*-*cas3*-*cas4*-*cas1*-*cas2*) (Fig. 3a). An additional 8 spacers flanked by the same direct repeat sequence were found elsewhere in the genome, yet were not associated with putative Cas-encoding genes. The presence of *cas3* and *cas8b* signature genes led to classification of this CRISPR-Cas locus within the Type I-B subtype.

We used BLAST^58^ and PHAST^59^ to analyze all 45 spacer tags specified in the *C. pasteurianum* genome in an attempt to identify protospacer matches from invading nucleic acid elements, including phages, prophages, plasmids, and transposons. Since seed sequences, rather than full-length protospacers, have been shown to guide CRISPR interference^60^, mismatches in the PAM-distal region of protospacer were permitted, while spacer-protospacer matches possessing more than one mismatch in 7 nt of PAM-proximal seed sequence were omitted. Although no perfect spacer-protospacer matches were identified, several hits were revealed possessing 2–7 mismatches to full-length *C. pasteurianum* spacers (Table 1). All protospacer hits identified were represented by spacers 18, 24, and 30 from the central *C. pasteurianum* Type I-B CRISPR array, whereby multiple protospacer hits were obtained using spacers 24 and 30. Importantly, protospacer matches were derived from predicted *Clostridium* and *Bacillus* phage and prophage elements.

### Probing the *C. pasteurianum* Type I-B CRISPR-Cas system using *in vivo* interference assays and elucidation of protospacer adjacent motif (PAM) sequences

We selected the best protospacer hits, possessing 2–4 nt mismatches to *C. pasteurianum* spacers 18, 24, and 30 (Table 1), for further characterization. Previous analyses of Type I CRISPR-Cas systems have employed a 5 nt mismatch threshold for identifying putative spacer-protospacer hits^26^,^61^, as imperfect pairing affords flexibility in host recognition of invading elements or indicates evolution of invading protospacer sequences as a means of evading CRISPR attack^60^. While the top spacer 30 hit was found to possess homology to an intact prophage from *C. botulinum*, the best spacer 24 match was predicted to target clostridial phage φCD111, a member of the *Siphoviridae* phage family. *C. pasteurianum* has recently been shown to harbor an intact and excisable temperate prophage from the same phage family, further supporting the notion that spacer 24 targets phage φCD111. The single protospacer match to spacer 18 was found to possess homology to a partial prophage region within the genome of *C. pasteurianum* BC1, a distinct strain from the type strain (ATCC 6013) employed in this study. Based on these analyses, it is probable that the phage and prophage elements described above are recognized by the *C. pasteurianum* Type I-B CRISPR-Cas machinery.

**Figure 1:**
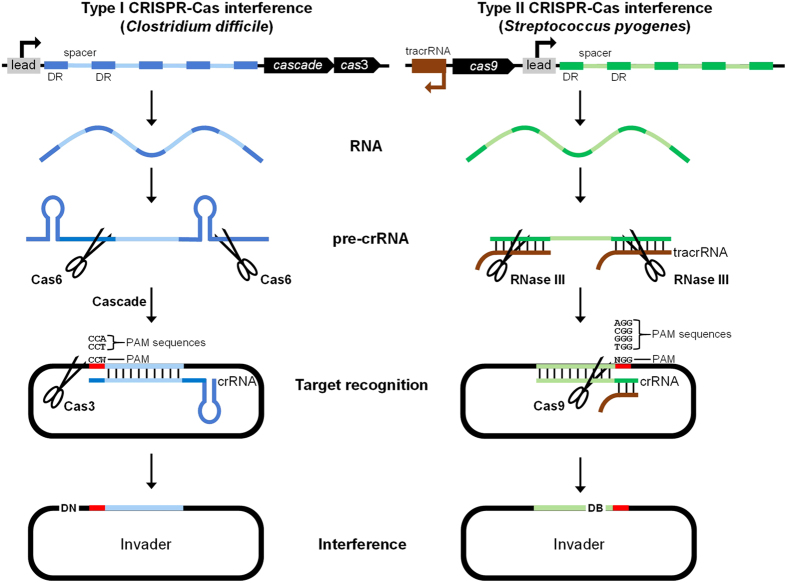
Comparison of Type I (left) and Type II (right) CRISPR-Cas interference mechanisms. CRISPR arrays, comprised of direct repeats (DRs; royal blue and dark green) and spacer tags (light blue and light green) are first transcribed into a single large pre-crRNA by a promoter located within the CRISPR leader (lead). The resulting transcript is cleaved and processed into individual mature crRNAs by the Cas6 endonuclease (Type I systems) or the ubiquitous RNase III enzyme (Type II systems). Processing is mediated by characteristic secondary structures (hairpins) formed by Type I pre-crRNAs or by a trans-activating RNA (tracrRNA; brown) possessing homology to direct repeat sequences in Type II systems. A single synthetic guide RNA (gRNA) can replace the dual crRNA-tracrRNA interaction (not shown). Mature crRNAs are guided to invading nucleic acids through homology between crRNAs and the corresponding invader protospacer sequence. Type I interference requires the multiprotein Cascade complex (comprised of *cas6*-*cas8b*-*cas7*-*cas5* in *Clostridium difficile*^62^ and *C. pasteurianum*), encoded downstream of the Type I CRISPR array. Type I and II interference mechanisms require recognition of one of multiple protospacer adjacent motif (PAM) sequences, which collectively comprise the consensus PAM element (red). The location of the PAM and the site of nucleolytic attack relative to the protospacer sequence differs between Type I and II CRISPR-Cas systems. Representative PAM sequences from *C. difficile* (Type I-B)^62^ and *Streptococcus pyogenes* (Type II)^25^ CRISPR-Cas loci are shown. Nucleolytic attack by Cas3 or Cas9 results in a DNA nick (DN) or blunt double-stranded DNA break (DB), respectively. Both CRISPR-Cas loci contain *cas1* and *cas2* genes (not shown), while the Type I and II loci also contain *cas4* and *csn2* genes, respectively (not shown).

Spacers 18, 24, and 30 were utilized to assess activity of the *C. pasteurianum* Type I-B CRISPR-Cas system using plasmid transformation interference assays. *C. pasteurianum* spacer sequences, rather than the identified protospacer hits possessing 2–4 mismatches, were utilized as protospacers to ensure 100% identity between *C. pasteurianum* spacers and plasmid-borne protospacers. As Type I and II CRISPR-Cas systems require the presence of a PAM sequence for recognition of invading elements^24^,^25^, a protospacer alone is not sufficient to elicit attack by host Cas proteins. Moreover, PAM elements are typically species-specific and vary in length, GC content, and degeneracy^26^. Accordingly, PAMs are often determined empirically and cannot be directly inferred from protospacer sequences. Hence, we constructed four derivatives each of protospacers 18, 24, and 30, yielding 12 constructs in total, whereby each protospacer was modified to contain different combinations of protospacer-adjacent sequence. Protospacer-adjacent sequences were derived from nucleotide sequences upstream or downstream of the protospacer matches within the DNA of the invading phage determinants depicted in Table 1. Five nt of protospacer-adjacent sequence was selected on the basis that most PAMs are encompassed within 5 nt^26^. Specifically, each protospacer derivative was constructed with one of four protospacer-adjacent sequence arrangements: 1) no protospacer-adjacent sequences; 2) 5 nt of 5′ protospacer-adjacent sequence; 3) 5 nt of 3′ protospacer-adjacent sequence; and 4) 5 nt of 5′ and 3′ protospacer-adjacent sequence (Fig. 3b). Although the PAM element is typically located at the 5′ end of protospacers in Type I CRISPR-Cas systems, which is opposite to the arrangement observed in Type II systems^26^ (Fig. 1), we elected to assay both 5′ and 3′ protospacer-adjacent sequences in the event that the *C. pasteurianum* Type I-B machinery exhibits atypical PAM recognition. Protospacer derivatives were synthesized as complementary single-stranded oligonucleotides, which were annealed and inserted into plasmid pMTL85141. Interestingly, all three protospacers triggered an interference response from *C. pasteurianum* when a suitable protospacer-adjacent sequence was provided (Fig. 3b). Plasmids devoid of 5′ protospacer-adjacent sequence (pSpacer18, pSpacer24, pSpacer30, pSpacer18-3′, pSpacer24-3′, and pSpacer30-3′), efficiently transformed *C. pasteurianum* (1.0–2.4 × 10^3^ CFU μg^−1^ DNA) (Fig. 3b). Conversely, plasmids containing 5′ protospacer-adjacent sequence (pSpacer18-5′, pSpacer24-5′, pSpacer30-5′, pSpacer18-flank, pSpacer24-flank, and pSpacer30-flank), were unable to transform *C. pasteurianum* (Fig. 3b). These data indicate that *C. pasteurianum* expresses Cas proteins that recognize specific PAM sequences encompassed within 5 nt at the 5′ end of protospacers. Interference by host Cas proteins was found to be robust and highly specific.

We analyzed the 5′-adjacent sequences corresponding to protospacers 18, 24, and 30, resulting in three functional PAM sequences represented by 5′-TTTCA-3′, 5′-AATTG-3′, and 5′-TATCT-3′, respectively (Fig. 3b and Table 1). Due to the promiscuity of most PAM elements, the identified PAM sequences presumably represent only a small subset of sequences that together constitute the consensus recognized by *C. pasteurianum*. It is noteworthy, however, that the third nucleotide of all three functional PAM sequences, as well as six additional sequences that were not assayed *in vivo* (Table 1), represents a conserved thymine (T) residue, which may be essential for recognition of invading determinants by *C. pasteurianum* Cas proteins. Within protospacer constructs lacking 5′ adjacent sequence, namely pSpacer18, pSpacer24, pSpacer30, pSpacer18-3′, pSpacer24-3′, and pSpacer30-3′, protospacers are preceded by the sequence 5′-CCGCG-3′ or 5′-CGCGG-3′, encompassing the partial SacII cloning site. It is evident that this sequence does not constitute a PAM sequence recognized by *C. pasteurianum* CRISPR-Cas machinery (Fig. 3b). Similarly, in their native context within the chromosome of *C. pasteurianum*, spacers 18, 24, and 30 are preceded by the sequence 5′-TAAAT-3′, which is also not recognized by host Cas proteins in order to avoid self attack. Although this sequence resembles the three functional PAM sequences identified through interference assays, particularly 5′-TATCT-3′, the central conserved T nucleotide is lacking, further supporting the importance of this residue in self and non-self distinction by *C. pasteurianum*.

By assuming the PAM sequence recognized by *C. pasteurianum* is 5 nt in length and based on a *C. pasteurianum* chromosomal GC content of 30%, it is possible to calculate the frequency that each PAM sequence occurs within the genome of *C. pasteurianum*. All three 5 nt *C. pasteurianum* PAM sequences are comprised of four A/T residues and one G/C residue, indicating that all PAM sequences should occur at the same frequency within the *C. pasteurianum* chromosome. Since the probability of an A or T nucleotide occurring in the genome is 0.35 and the probability of a C or G nucleotide is 0.15, the frequency of each PAM sequence within either strand of the *C. pasteurianum* genome is 1 ÷ [(0.35)^4^(0.15)(2 strands)] = 222 bp. More importantly, the overall PAM frequency is only 74 bp, indicating that one of the three functional PAM sequences is expected to occur every 74 bp within the genome of *C. pasteurianum*. This frequency is further reduced to 27 bp if the true PAM recognized by *C. pasteurianum* is represented by 3 nt, which is a common feature of Type I-B PAMs^62^,^63^. In comparison, the Type II CRISPR-Cas9 system from *S. pyogenes* recognizes a 5′-NGG-3′ consensus, which is expected to occur every 22 bp in the genome of *C. pasteurianum*.

### Repurposing the endogenous Type I-B CRISPR-Cas system for markerless genome editing

The high frequency of functional PAM sequences within the genome of *C. pasteurianum* suggests that the endogenous Type I-B CRISPR-Cas system could be co-opted to attack any site within the organism’s chromosome and, therefore, provide selection against unmodified host cells. To first assess self-targeting of the *C. pasteurianum* CRISPR-Cas system, we again selected the *cpaAIR* gene as a target. The 891 bp *cpaAIR* gene was found to possess a total of 19 potential PAM sequences (5′-TTTCA-3′, 5′-AATTG-3′, and 5′-TATCT-3′), which is more than the 12 PAM sequences expected based on a genomic frequency of 74 bp. We selected one PAM sequence (5′-AATTG-3′) within the coding region of the *cpaAIR* gene as the target site for *C. pasteurianum* self-cleavage, whereby sequence immediately downstream embodies the target protospacer. Analysis of the core 37 spacers encoded by *C. pasteurianum* revealed minimal variation in spacer length (34–37 nt; mean of 36 nt), while GC content was found to vary dramatically (17–44%). Subsequently, we generated a synthetic *cpaAIR* spacer by selecting 36 nt immediately downstream of the designated PAM sequence, which was found to possess a GC content of 28%. A CRISPR expression cassette was designed by mimicking the sequence and arrangement of the native Type I-B CRISPR array present in the *C. pasteurianum* genome (Figure S1B). Specifically, a 243 bp CRISPR leader was utilized to drive transcription of the synthetic *cpaAIR* CRISPR array, comprised of the 36 nt *cpaAIR* spacer flanked by 30 nt direct repeats. The synthetic array was followed by 298 bp of sequence located at the 3′ end of the endogenous chromosomal CRISPR array. The resulting cassette was synthesized and inserted into plasmid pMTL85141, generating pCParray-cpaAIR (Fig. 4a). While several attempts at transformation of *C. pasteurianum* using pCParray-cpaAIR failed to generate transformants, an overall transformation efficiency of 0.6 CFU μg^−1^ DNA was obtained (Fig. 4b), compared to 6.3 × 10^3^ CFU μg^−1^ DNA for the pMTL85141 parental plasmid, a difference of more than four orders of magnitude. We reasoned that the synthetic *cpaAIR* spacer triggered self-attack of *C. pasteurianum* through introduction of a DN and subsequent strand degradation by Cas3. To verify the location of the DN site within the *cpaAIR* target gene and, more importantly, demonstrate manipulation of the Type I-B CRISPR-Cas system for genome editing, we introduced the aforementioned *cpaAIR* editing cassette utilized for *cas9*-mediated genome editing (from plasmid pCas9gRNA-delcpaAIR) into plasmid pCParray-cpaAIR (Fig. 4a). Transformation of *C. pasteurianum* with the resulting plasmid, pCParray-delcpaAIR, produced an abundance of transformants, yielding a transformation efficiency of 9.5 CFU μg^−1^ DNA, an increase of more than an order of magnitude compared to pCParray-cpaAIR lacking an editing cassette (Fig. 4b). Despite a low-level of background resulting from transformation with pCParray-cpaAIR, genotyping of 10 pCParray-delcpaAIR transformants generated a PCR product corresponding to *cpaAIR* gene deletion in all colonies screened, yielding an editing efficiency of 100% (Fig. 4c). Sanger sequencing of a single pCParray-delcpaAIR transformant confirmed successful deletion of a 762 bp region of the *cpaAIR* coding sequence (data not shown). Importantly, this outcome is consistent with localization of the DN within the *cpaAIR* locus, as well as provides proof-of-principle repurposing of the host Type I-B CRISPR-Cas machinery for efficient markerless genome editing.

### Identification of putative PAM sequences in industrial and pathogenic clostridia

As the first step towards expanding our CRISPR-Cas hijacking strategy to other prokaryotes, we surveyed the clostridia for species harboring putative CRISPR-Cas loci. One cellulolytic and one acetogenic species, namely *Clostridium thermocellum* and *Clostridium autoethanogenum*, respectively, in addition to *Clostridium tetani*, a human pathogen, were selected. Like *C. pasteurianum*, all three species encode putative Type I-B systems, while *C. tetani*^64^ and *C. thermocellum*^65^ harbor an additional Type I-A or Type III locus, respectively. Only spacers associated with Type I-B loci were analyzed, corresponding to 98, 31, and 169 spacers from *C. autoethanogenum*, *C. tetani*, and *C. thermocellum*, respectively. *In silico* analysis of clostridial spacers against firmicute genomes, phages, and plasmids yielded putative protospacer matches from all three clostridial Type I-B CRISPR-Cas loci analyzed (Table 2). In total 10 promising protospacer hits were obtained, which were found to target phages (2 hits), plasmids (1 hit), predicted prophages (5 hits), and regions of bacterial genomes in the vicinity of phage and/or transposase genes (2 hits). Six spacers were found to target clostridial genomes and clostridial phage and prophage elements. Interestingly, spacers from the *C. autoethanogenum* Type I-B locus were analyzed in an earlier report and no putative protospacer matches were identified^65^, whereas we unveiled four probable protospacer hits, including the only perfect spacer-protospacer match identified in this study. Overall, putative protospacer matches contained 0–8 mismatches when aligned with clostridial spacers. Analysis of clostridial 5′-protospacer-adjacent sequences revealed a number of conserved sequences (Table 2). Interestingly, all 10 putative PAM sequences were found to possess a conserved A residue in the immediate 5′ protospacer-adjacent position. Based on a 3 nt consensus, prospective PAMs of 5′-NAA-3′ (PAM sequences: 5′-CAA-3′, 5′-GAA-3′, 5′-TAA-3′, and 5′-TAA-3′), 5′-TNA-3′ (PAM sequences: 5′-TAA-3′, 5′-TCA-3′, and 5′-TTA-3′), and 5′-NCA-3′ (PAM sequences: 5′-ACA-3′, 5′-TCA-3′, 5′-TCA-3′) could be predicted for the Type I-B CRISPR-Cas loci of *C. autoethanogenum*, *C. tetani*, and *C. thermocellum*, respectively.

## Discussion

This work details the development of a genome editing methodology allowing efficient introduction of precise chromosomal modifications through harnessing an endogenous CRISPR-Cas system. Our strategy leverages the widespread abundance of prokaryotic CRISPR-Cas machinery, which have been identified in 45% of bacteria, including 74% of clostridia^7^. An exceptional abundance of CRISPR-Cas loci, coupled with an overall lack of sophisticated genetic engineering technologies and tremendous biotechnological potential, provides the rationale for our proposed genome editing strategy in *Clostridium*. We selected *C. pasteurianum* for proof-of-concept CRISPR-Cas repurposing due to the presence of a Type I-B CRISPR-Cas locus (Fig. 3a) and established industrial relevance for biofuel production^48^,^66^. Analysis of *C. pasteurianum* CRISPR tags led to elucidation of the probable origins of three spacer sequences, all of which returned protospacer matches from clostridial phage and prophage determinants (Table 1). *C. pasteurianum* Cas proteins proved to be functional and highly active against plasmid-borne protospacers possessing a 5′ adjacent PAM sequence, as no interference response was generated from protospacers harboring 3′ adjacent sequence in the absence of a 5′ PAM sequence (Fig. 3b). This finding is consistent with other Type I CRISPR-Cas systems, in which the PAM positioned 5′ to the protospacer is essential for interference by host cells and contrasts Type II CRISPR-Cas9 systems, whereby the PAM is recognized at the 3′ end of protospacers^14^,^25^,^26^. Following elucidation of functional PAM sequences, we developed a genome editing strategy encompassing expression of a synthetic programmable Type I-B CRISPR array that guides site-specific nucleolytic attack of the *C. pasteurianum* chromosome by co-opting the organism’s native Cas proteins. Cas3-mediated DNA attack affords selection against unmodified host cells, whereby edited cells are efficiently obtained through co-introduction of an editing template (Fig. 4a,b). We have demonstrated 100% editing efficiency (10/10 correct colonies) by targeting the *cpaAIR* locus in combination with introduction of a *cpaAIR* gene deletion cassette (Fig. 4c).

**Figure 2:**
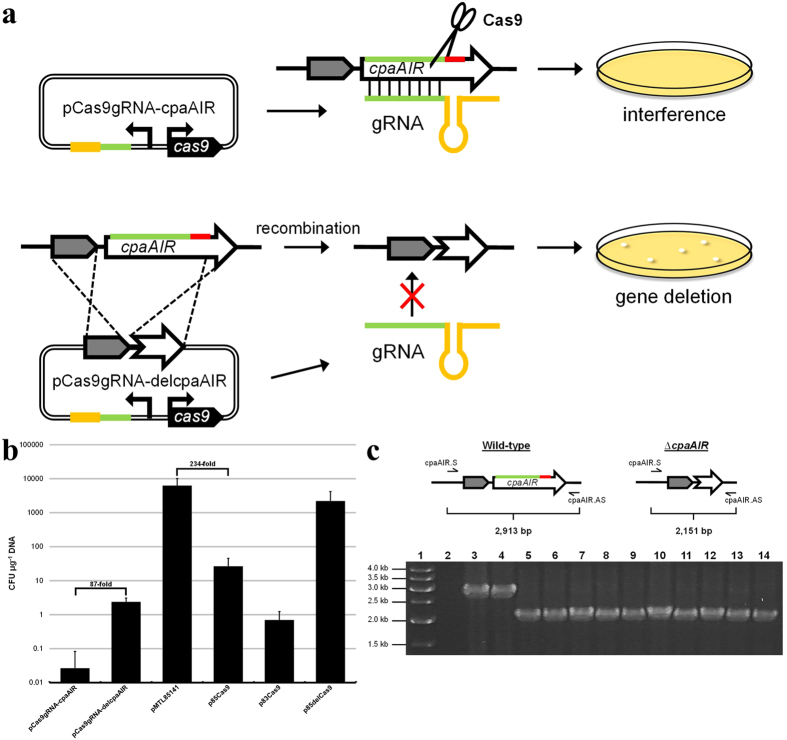
Genome editing in *C. pasteurianum* using the heterologous *S. pyogenes* Type II CRISPR-Cas9 system. **(a)** *cpaAIR* gene deletion strategy using Type II CRISPR-Cas9. Introduction of a double-stranded DB to the *cpaAIR* locus was achieved by programming a gRNA spacer sequence (green) and expressing heterologous *cas9* within plasmid pCas9gRNA-cpaAIR. *cpaAIR*-targeted gRNA, containing *cas9* binding handle (orange), is directed to the chromosomal *cpaAIR* gene through base-pairing to the protospacer sequence and Cas9-recognition of the *S. pyogenes* PAM element (5′-NGG-3′; red). Insertion of a *cpaAIR* gene editing cassette in pCas9gRNA-cpaAIR, generating pCas9gRNA-delcpaAIR, leads to homologous recombination and deletion of a portion of the *cpaAIR* coding sequence, including the protospacer and PAM elements. Unmodified cells are selected against by Cas9 cleavage, while edited cells possessing a partial *cpaAIR* deletion are able evade attack. Genes, genomic regions, and plasmids are not depicted to scale. **(b)** Transformation efficiency corresponding to Type II CRISPR-Cas9 vectors (pCas9gRNA-cpaAIR and pCas9gRNA-delcpaAIR) and various *cas9* expression derivatives and control constructs (pMTL85141, p85Cas9, p83Cas9, p85delCas9). Transformation efficiency is reported as the number of CFU generated per μg of plasmid DNA. Data shown are averages resulting from at least two independent experiments and error bars depict standard deviation. **(c)** Colony PCR genotyping of pCas9gRNA-delcpaAIR transformants. Primers cpaAIR.S and cpaAIR.AS were utilized in colony PCR to screen 10 colonies harboring pCas9gRNA-delcpaAIR. Expected product sizes are shown corresponding to the wild-type (2,913 bp) and the *cpaAIR* deletion mutant (2,151 bp) strains of *C. pasteurianum*. Lane 1: linear DNA marker; lane 2: no colony control; lanes 3: wild-type colony; 4: colony harboring pCas9gRNA-cpaAIR; lanes 5–14: colonies harboring pCas9gRNA-delcpaAIR.

**Figure 3:**
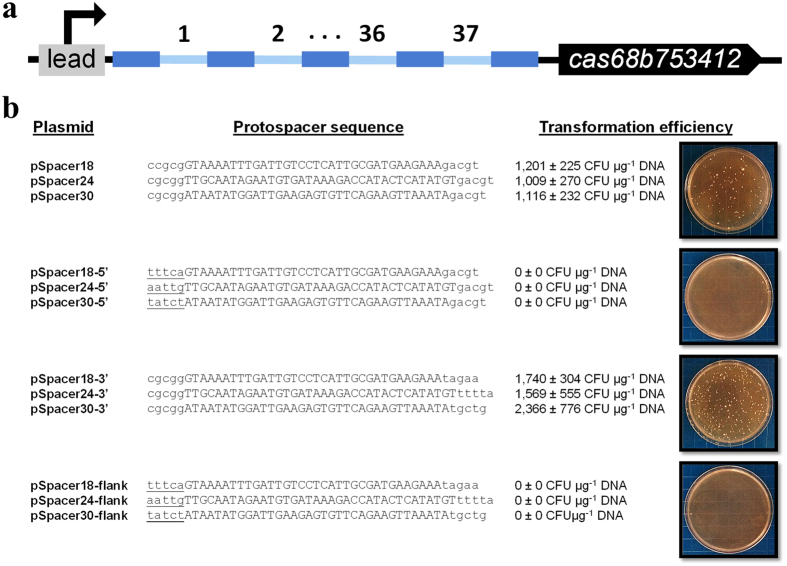
Characterization of the central Type I-B CRISPR-Cas system of *C. pasteurianum*. **(a)** Genomic structure of the Type I-B CRISPR-Cas locus of *C. pasteurianum*. The central CRISPR-Cas locus is comprised of 37 distinct spacers (light blue) flanked by 30 nt direct repeats (royal blue) and a representative Type I-B *cas* operon containing *cas6*-*cas8b*-*cas7*-*cas5*-*cas3*-*cas4*-*cas1*-*cas2* (abbreviated *cas68b753412*). A promoter within the putative leader sequence (lead) drives transcription of the CRISPR array. **(b)** Plasmid interference assays using protospacers 18, 24, and 30 (uppercase) and different combinations of 5′ and/or 3′ protospacer-adjacent sequence (lowercase). Protospacers were designed to possess no adjacent sequences, 5′ or 3′ adjacent sequence, or both 5′ and 3′ adjacent sequences. Protospacers were cloned in plasmid pMTL85141 and the resulting plasmids were used to transform *C. pasteurianum*. Putative PAM sequences are underlined. Pictures of representative transformants are shown corresponding to protospacer 30.

**Figure 4:**
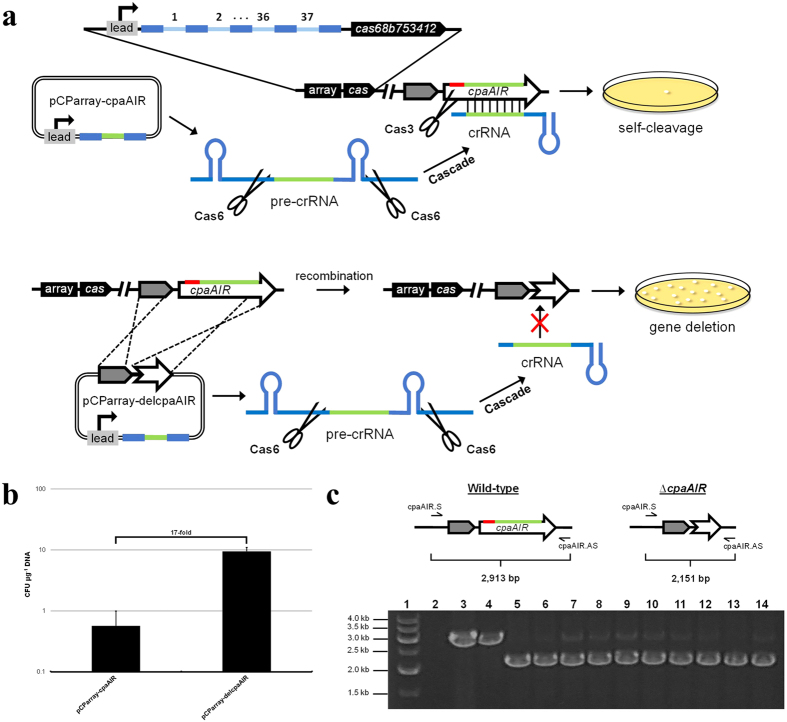
Genome editing in *C. pasteurianum* using the endogenous Type I-B CRISPR-Cas system. **(a)** *cpaAIR* gene deletion strategy using endogenous Type I-B CRISPR-Cas machinery. A condensed *C. pasteurianum* Type I-B CRISPR array (array) and *cas* gene operon (*cas*) is shown, in addition to the *cpaAIR* targeting locus. An inset is provided showing the full-length *C. pasteurianum* CRISPR-Cas locus comprised of a 37-spacer array and *cas* operon containing *cas6*-*cas8b*-*cas7*-*cas5*-*cas3*-*cas4*-*cas1*-*cas2* (abbreviated *cas68b753412*). Introduction of a DNA nick to the *cpaAIR* gene was achieved by expressing a synthetic CRISPR array containing a 36 nt *cpaAIR* spacer (green) flanked by 30 nt direct repeats (royal blue) within plasmid pCParray-cpaAIR. The synthetic array is transcribed into pre-crRNA and processed into mature crRNA by Cas6. crRNA processing and interference occurs as depicted in Fig. 1. In some experiments, selection against wild-type cells using pCParray-cpaAIR generated a single background colony. Insertion of a *cpaAIR* gene editing cassette in pCParray-cpaAIR, generating pCParray-delcpaAIR, leads to homologous recombination and deletion of a portion of the *cpaAIR* coding sequence, including the protospacer and PAM sequence (5′-AATTG-3′). Unmodified cells are selected against by Cas3 cleavage, while edited cells possessing a partial *cpaAIR* deletion are able to survive. Genes, genomic regions, and plasmids are not depicted to scale. **(b)** Transformation efficiency corresponding to Type I-B CRISPR-Cas vectors. Transformation efficiency is reported as the number of CFU generated per μg of plasmid DNA. Data shown are averages resulting from at least two independent experiments and error bars depict standard deviation. **(c)** Colony PCR genotyping of pCParray-delcpaAIR transformants. Primers cpaAIR.S and cpaAIR.AS were utilized in colony PCR to screen 10 colonies harboring pCParray-delcpaAIR. Expected product sizes are shown corresponding to the wild-type (2,913 bp) and the *cpaAIR* deletion mutant (2,151 bp) strains of *C. pasteurianum*. Lane 1: linear DNA marker; lane 2: no colony control; lanes 3: wild-type colony; 4: colony harboring pCParray-cpaAIR; lanes 5–14: colonies harboring pCParray-delcpaAIR.

Our native CRISPR-Cas repurposing methodology contrasts current approaches of CRISPR-mediated genome editing in bacteria, which rely on the widely-employed Type II CRISPR-Cas9 system from *S. pyogenes*. In *Clostridium*, such heterologous CRISPR-Cas9 genome editing strategies have recently been implemented in *C. beijerinckii*^46^ and *C. cellulolyticum*^47^. While editing efficiencies >95% were reported using *C. cellulolyticum*, no efficiency was provided for CRISPR-based editing in *C. beijerinckii*, which involves the use of a phenotypic screen to identify mutated cells^46^. Although we have shown 100% editing efficiency in *C. pasteurianum* through application of the same *S. pyogenes* CRISPR-Cas9 machinery (Fig. 2a,c), the total yield of edited cells was only 25% compared to the endogenous Type I-B CRISPR-Cas approach (Figs 2b and 4b). By assessing transformation of various *cas9* expression constructs, we ascribe this outcome to poor transformation of vectors expressing *cas9 in trans* (Fig. 2b). A low to moderate level of Cas9 toxicity has been documented in a diverse range of organisms, including protozoa^67^, *Drosophila*^68^,^69^, yeast^54^, mice^32^, and human cells^70^, and likely results from the generation of lethal ectopic chromosomal DNA breaks. We have also observed reduced transformation of *E. coli* ER1821 in this study using plasmids expressing heterologous *cas9* (data not shown). In more dramatic instances, for example in mycobacteria^56^ and the alga *Chlamydomonas reinhardtii*^55^, toxicity leads to erratic *cas9* expression and overall poor genome editing outcomes. Such reports emphasize the importance of mitigating Cas9 toxicity or developing alternative methodologies facilitating efficient genome editing^55^. Owing to the notoriously low transformation efficiencies achieved using *Clostridium* species (typically 10^2^–10^3^ CFU μg^−1^ DNA)^41^, the clostridia are especially susceptible to the detrimental effects of heterologous *cas9* expression, as observed in this study. Hence, for key organisms lacking endogenous CRISPR-Cas loci, such as *C. acetobutylicum* and *C. ljungdahlii*, in which the heterologous Type II system is obligatory for genome editing, we recommend inducible expression of *cas9*. For this purpose, several clostridial inducible gene expression systems have recently been characterized^71^,^72^. Our success in obtaining targeted mutants using constitutive expression of heterologous *cas9* potentially results from the relatively high efficiency of plasmid transfer to *C. pasteurianum* (up to 10^4^ CFU μg^−1^ DNA)^49^. It is probable that Cas9-mediated genome editing efforts could be impeded in species that are poorly transformed, rendering endogenous CRISPR-Cas machinery the preferred platform for genome editing. Furthermore, since linear DNA is a poor substrate for transformation of *Clostridium* and because it is generally unfeasible to co-transfer two DNA substrates to *Clostridium* due to poor transformation, all of the genetic components required for Type I-B or Type II CRISPR-Cas functionality in this study were expressed from single vectors. This shortcoming exposes an additional advantage of our endogenous CRISPR-Cas hijacking strategy, as only a small CRISPR array (0.6 kb) and editing template are required for genome editing, resulting in a compact 5.7 kb editing vector (pCParray-delcpaAIR). On the other hand, editing using the heterologous Type II system requires expression of the large 4.2 kb *cas9* gene, in addition to a 0.4 kb gRNA cassette and editing template. The large size of the resulting pCas9gRNA-delcpaAIR editing vector (9.7 kb) not only limits transformation but also places significant constraints on multiplexed editing strategies involving multiple gRNAs and editing templates. Owing to overall low rates of homologous recombination in *Clostridium*, such ambitious genome editing strategies could be enhanced through coupling of native or heterologous CRISPR-Cas machinery to highly recombinogenic phage activities^73^. In this context, one functional clostridial phage recombinase has been characterized to date^74^.

To initiate efforts aimed at co-opting Type I CRISPR-Cas machinery in other key species, we examined CRISPR spacer tags from one acetogenic (*C. autoethanogenum*), one cellulolytic (*C. thermocellum*), and one pathogenic (*C. tetani*) species (Table 2). Subsequent *in silico* analysis of clostridial spacers, coupled with our experimental validation of *C. pasteurianum* PAM sequences and a recent report detailing characterization of the *C. difficile* Type I-B CRISPR-Cas locus^62^, provide an in depth glimpse into clostridial CRISPR-Cas defence mechanisms (Table 3). Overall, clostridial Type I-B PAM sequences are characterized by a notable lack of guanine (G) residues. Additionally, several PAM sequences unveiled in this study are recognized across multiple species of *Clostridium*, such as 5′-TCA-3′ by *C. pasteurianum*, *C. tetani*, and *C. thermocellum*, and 5′-TAA-3′ by *C. autoethanogenum* and *C. tetani*, which suggests horizontal transfer of CRISPR-Cas loci between these organisms. Indeed, *C. tetani* harbors 7 distinct Type I-B CRISPR arrays^64^, 3 of which employ the same direct repeat sequence utilized by the *C. pasteurianum* Type I-B system. Since PAM sequences determined in this study are highly similar between *C. pasteurianum* (5′-TCA-3′, 5′-TTG-3′, 5′-TCT-3′) and *C. tetani* (5′-TCA-3′, 5′-TTA-3′, 5′-TAA-3′), it is plausible that these organisms recognize the same PAM consensus. More broadly, clostridial Type I-B PAM sequences bear a striking overall resemblance to sequences recognized by the Type I-B system from the distant archaeon *Haloferax volcanii* (5′-ACT-3′, 5′-TTC-3′, 5′-TAA-3′, 5′-TAT-3′, 5′-TAG-3′, and 5′-CAC-3′)^63^, which are also distinguished by an overall low frequency of G residues. Collectively these data suggest that many PAM sequences are common amongst Type I-B CRISPR-Cas systems, even in evolutionarily distant species, such as the case of *Haloferax* and *Clostridium*. In this context, we posit that empirical elucidation of PAMs is unnecessary, as highly pervasive PAM sequences (e.g., 5′-TCA-3′ and 5′-TAA-3′) or validated sequences from closely-related species can easily be assessed for functionality in a target host strain. This consequence simplifies our proposed CRISPR-Cas repurposing approach, as a functional PAM sequence and a procedure for plasmid transformation are the only prerequisite criteria for implementing our methodology in any target organism harboring active Type I CRISPR-Cas machinery.

Genome editing strategies based on the *S. pyogenes* Type II system reported previously^46^,^47^ and the CRISPR-Cas hijacking approach detailed in this study, represent a key divergence from earlier methods of gene disruption and integration in *Clostridium*^41^. Currently, the only procedures validated for modifying the genome of *C. pasteurianum* involve the use of a programmable group II intron^50^ and heterologous counter-selectable *mazF* marker^75^. Whereas group II introns are limited to gene disruption, as deletion and replacement are not possible, techniques based on homologous recombination using antibiotic resistance determinants and counter-selectable markers, such as *pyrE*/*pyrF*, *codA*, and *mazF*^42^,^43^,^76^, are technically-challenging and laborious due to a requirement for excision and recycling of markers. In general, these strategies do not provide adequate selection against unmodified cells, necessitating subsequent rounds of enrichment and selection^42^,^43^,^76^,^77^. Thus, both native and heterologous CRISPR-Cas machineries offer more robust platforms for genome modification of *C. pasteurianum* and related clostridia.

Currently, endogenous CRISPR-Cas systems have been harnessed in only a few prokaryotes, namely *E. coli*^78^,^79^, *Pectobacterium atrosepticum*^80^, *Streptococcus thermophilus*^78^, and two species of archaea^52^,^81^. In conjunction with these reports, our success in co-opting the chief *C. pasteurianum* CRISPR-Cas locus contributes to a growing motivation towards harnessing host CRISPR-Cas machinery in a plethora of prokaryotes. The general rationale of endogenous CRISPR-Cas repurposing is not limited to genome editing, as a range of applications can be envisioned. In a recent example, Luo *et al*.^79^ deleted the native *cas3* endonuclease gene from *E. coli*, effectively converting the host Type I-E CRISPR-Cas immune system into a robust transcriptional regulator for gene silencing. Such applications dramatically extend the existing molecular genetic toolbox and pave the way to advanced strain engineering technologies. Although our work here focused on *C. pasteurianum*, repurposing of endogenous CRISPR-Cas loci is readily adaptable to most of the genus *Clostridium*, including many species of immense relevance to medicine, energy, and biotechnology, as well as half of all bacteria and most archaea.

## Materials and Methods

### Strains, plasmids, and oligonucleotides

Strains and plasmids employed in this study are listed in Table 4. *Clostridium pasteurianum* ATCC 6013 was obtained from the American Type Culture Collection (ATCC; Manassas, VA) and propagated and maintained according to previous methods^49^,^50^. *Escherichia coli* strains DH5α and ER1821 (New England Biolabs; Ipswich, MA) were employed for plasmid construction and plasmid methylation, respectively. Recombinant strains of *C. pasteurianum* were selected using 10 μg ml^−1^ thiamphenicol and recombinant *E. coli* cells were selected using 30 μg ml^−1^ kanamycin or 30 μg ml^−1^ chloramphenicol. Antibiotic concentrations were reduced by 50% for selection of double plasmid recombinant cells. Desalted oligonucleotides and synthetic DNA constructs were purchased from Integrated DNA Technologies (IDT; Coralville, IA). Oligonucleotides utilized in this study are listed in Table S1 and synthetic DNA constructs are detailed in Figure S1.

### DNA manipulation, plasmid construction, and transformation

A *cas9 E. coli*-*Clostridium* expression vector, p85Cas9, was constructed through amplification of a *cas9* gene cassette from pCas9^27^ using primers cas9.SacII.S + cas9.XhoI.AS and insertion into the corresponding sites of pMTL85141^57^. To construct an *E. coli*-*C. pasteurianum* Type II CRISPR-Cas9 plasmid (pCas9gRNA-cpaAIR) based on the *S. pyogenes* CRISPR-Cas9 system, we designed a synthetic gRNA cassette targeted to the *C. pasteurianum cpaAIR* gene by specifying a 20 nt *cpaAIR* spacer sequence (ctgatgaagctaatacagat), which was expressed from the *C. beijerinckii* sCbei_5830 small RNA promoter^46^. A promoter from the *C. pasteurianum* thiolase gene was included for expression of *cas9*. The resulting 821 bp DNA fragment (Figure S1A) was synthesized and inserted into the SacII and BstZ17I sites of p85Cas9. To modify pCas9gRNA-cpaAIR for genome editing via deletion of *cpaAIR*, splicing by overlap extension (SOE) PCR was utilized to fuse 1,028 bp and 1,057 bp *cpaAIR* homology regions generated using the primer sets delcpaAIR.PvuI.S + delcpaAIR.SOE.AS and delcpaAIR.SOE.S + delcpaAIR.PvuI.AS, respectively. The resulting PvuI-digested product was cloned into the PvuI site of pCas9gRNA-cpaAIR, yielding pCas9gRNA-delcpaAIR. Plasmid p83Cas9, a p85Cas9 derivative containing the pCB102 replication module, was constructed by amplifying *cas9* from pCas9^27^ using primers cas9.SacII.S + cas9.XhoI.AS and inserting the resulting product into the corresponding sites of pMTL83151^57^. A promoterless *cas9* derivative of p85Cas9, designated p85delCas9, was derived by amplification of a partial promoterless *cas9* fragment from pCas9gRNA-cpaAIR using primers –cas9.SacII.S + cas9.BstZ17I.AS and cloning of the resulting product into the SacII + BstZ17I sites of p85Cas9.

*C. pasteurianum* protospacer constructs lacking protospacer-adjacent sequences were derived by annealing oligos spacer18.AatII.S + spacer18.SacII.AS (pSpacer18), spacer24.AatII.S + spacer24.SacII.AS (pSpacer24), or spacer30.AatII.S + spacer30.SacII.AS (pSpacer30). Protospacer constructs possessing 5′ or 3′ protospacer-adjacent sequences were prepared by annealing oligos spacer18-5′.AatII.S + spacer18-5′.SacII.AS (pSpacer18-5′), spacer18-3′.AatII.S + spacer18-3′.SacII.AS (pSpacer18-3′), spacer24-5′.AatII.S + spacer24-5′.SacII.AS (pSpacer24-5′), spacer24-3′.AatII.S + spacer24-3′.SacII.AS (pSpacer24-3′), spacer30-5′.AatII.S + spacer30-5′.SacII.AS (pSpacer30-5′), or spacer30-3′.AatII.S + spacer30-3′.SacII.AS (pSpacer30-3′). Protospacer constructs possessing 5′ and 3′ flanking protospacer-adjacent sequence were prepared by annealing oligos spacer18-flank.AatII.S + spacer18-flank.SacII.AS (pSpacer18-flank), spacer24-flank.AatII.S + spacer24-flank.SacII.AS (pSpacer24-flank), or spacer30-flank.AatII.S + spacer30-flank.SacII.AS (pSpacer30-flank). In all instances protospacer oligos were designed such that annealing generated AatII and SacII cohesive ends for ligation with AatII- + SacII-digested pMTL85141.

To construct the endogenous CRISPR array vector, pCParray-cpaAIR, a synthetic CRISPR array was designed containing a 243 bp CRISPR leader sequence and a 37 nt *cpaAIR* spacer flanked by 30 nt direct repeat sequences. The synthetic array was followed by 298 bp of sequence found downstream of the endogenous CRISPR array in the chromosome of *C. pasteurianum* to ensure design of the synthetic array mimics that of the native sequence. The resulting 667 bp fragment (Figure S1B) was synthesized and cloned into the SacI site of pMTL85141. A genome editing derivative of pCParray-cpaAIR for deletion of *cpaAIR* was derived by subcloning the PvuI-flanked *cpaAIR* deletion cassette from pCas9gRNA-delcpaAIR into pCParray-cpaAIR, yielding pCParray-delcpaAIR.

DNA manipulation was performed according to established methods^82^. Commercial kits for DNA purification and agarose gel extraction were obtained from Bio Basic Inc. (Markham, ON). Plasmids were introduced to *C. pasteurianum*^49^ and *E. coli*^82^ using established methods of electrotransformation. Prior to transformation of *C. pasteurianum*, *E. coli*-*C. pasteurianum* shuttle plasmids were first methylated in *E. coli* ER1821 by the M.FnuDII methyltransferase from plasmid pFnuDIIMKn^49^. One to 5 μg of plasmid DNA was utilized for transformation of *C. pasteurianum*, except for plasmids harboring CRISPR-Cas machinery (pCas9gRNA-cpaAIR, pCas9gRNA-delcpaAIR, pCParray-cpaAIR, and pCParray-delcpaAIR), in which 15–25 μg was utilized to enhance transformation. Transformation efficiencies reported represent averages of at least two independent experiments and are expressed as colony-forming units (CFU) per μg of plasmid DNA.

### Identification of putative protospacer matches to clostridial spacers

Clostridial spacers were utilized to query firmicute genomes, phages, transposons, and plasmids using BLAST. Parameters were optimized for somewhat similar sequences (BlastN)^58^. Putative protospacer hits were assessed based on the number and location of mismatches, whereby multiple PAM-distal mutations were tolerated, while protospacers containing more than one mismatch within 7 nt of PAM-proximal seed sequence were rejected^60^. Firmicute genomes possessing putative protospacer hits were analyzed for prophage content using PHAST^59^ and surrounding sequences were inspected for elements indicative of DNA mobility and invasion, such as transposons, transposases, integrases, and terminases.

## Additional Information

**How to cite this article**: Pyne, M. E. *et al*. Harnessing heterologous and endogenous CRISPR-Cas machineries for efficient markerless genome editing in *Clostridium*. *Sci. Rep*. **6**, 25666; doi: 10.1038/srep25666 (2016).

## Supplementary Material

Supplementary Information

## Figures and Tables

**Table 1:** Putative protospacer matches identified through *in silico* analysis of *C. pasteurianum* CRISPR spacers.

Spacer number	Spacer-protospacer match^a^	Invading element^b^	Mismatches	Putative PAM sequence^c^
18	GTAAAATTTGATTGTCCTCATTGCGATGAAGAAA ATAAAATTTGATTGCCCTCACTGTGATGAAGAAA	*Clostridium pasteurianum* BC1 (vicinity of phage genes)	4	**5**′**-TTTCA-3**′
24	TTGCAATAGAATGTGATAAAGACCATACTCATATGT TTGCAATAGAATGCGATAAAGACCATACACATATGT	*Clostridium* phage φCD211	2	**5**′**-AATTG-3**′
	TTGCAATAGAATGTGATAAAGACCATACTCATATGT TAGCAATAGAATGTGATAGAGATCATACGCATATGT	*Clostridium acidurici* 9a (transposase)	4	5′-AATTA-3′
	TTGCAATAGAATGTGATAAAGACCATACTCATATGT TGGCAATAGAATGTGATAAAGACCACTGCCATCTTT	*Clostridium aceticum* strain DSM 1496 plasmid CACET_5p (transposase)	7	5′-AATTT-3′
30	ATAATATGGATTGAAGAGTGTTCAGAAGTTAAATA ATAATATGGATAGAAGAATGTTCAGAAGTAAAATA	*Clostridium botulinum* CDC_297 (intact prophage)	3	**5**′**-TATCT-3**′
	ATAATATGGATTGAAGAGTGTTCAGAAGTTAAATA TTAATATGGATAGAAGAATGTTCAGAAGTTAAATA	*Clostridium pasteurianum* NRRL B-598 (intact prophage)	3	5′-TTTCT-3′
	ATAATATGGATTGAAGAGTGTTCAGAAGTTAAATA ATAATATGGATTGAGGAATGTTCAGAGGTCAAATA	*Bacillus licheniformis* ATCC 14580 (phage terminase)	4	5′-TCTCA-3′
	ATAATATGGATTGAAGAGTGTTCAGAAGTTAAATA ATCATATGGATTGAGGAATGTTCAGAAGTTAAGTA	*Bacillus pumilus* strain NJ-V2 (phage terminase)	4	5′-TCTCG-3′
	ATAATATGGATTGAAGAGTGTTCAGAAGTTAAATA TTAATATGGATTGAAGAGTGCTCAGAGGTGAAGTA	*Bacillus subtilis* strain SG6 (intact prophage)	5	5′-TTTCA-3′

^a^Spacer-protospacer mismatches are underlined.

^b^For hits found within bacterial genomes, the location of the protospacer sequence relative to prophage regions and mobile genetic elements is provided in parentheses.

^c^5 nt of adjacent sequence is provided. PAM sequences corresponding to the top protospacer hit from each spacer (bolded) were selected for *in vivo* interference assays.

**Table 2:** Putative protospacer matches identified through *in silico* analysis of clostridial CRISPR spacers.

Organism (CRISPR-Cas subtype)	Spacer-protospacer match^a^	Invading element^b^	Mismatches	Putative PAM sequence^c^
*C. autoethanogenum* DSM 10061 (Type I-B)	AAGAGTTGATACTTTACTTATAGATTACTTAGGTGCAAGAGTTGATACTTTACTTATAGATTACTTAGGTGC	*Clostridium ljungdahlii* DSM 13528 (incomplete prophage)	0	5′-ATT**AA**-3′
	TAGACCACAATTAAATGCAATGTTAGAATTTGCTCGTAGGCCACAATTAAAAGCCATGTTAGAATTTGCTAG	*Clostridium* phage vB_CpeS-CP51	4	5′-ACT**AA**-3′
	AAATACATTTTATAAATTATTAAAAGAATATGAGGAAATACTTTTTATAAAATATTGAAAGAATATGAAG	*Bacillus thuringiensis* HD-789 plasmid pBTHD789-3	4	5′-AAG**AA**-3′
	GCAGCTCCAGGAGCAAAAACCAAAGGTACTATTCGCGAAGCTCCAGGAGCAAAAATCAAAGGTATTTATTTT	*Enterococcus durans* strain KLDS 6.0930 (vicinity of transposase and phage genes)	8	5′-ATC**AA**-3′
*C. tetani* 12124569 (Type I-B)	ATATTTCTTTTTTACTCCAATAAGCTCCAATGAGATATTTCTTTTTTACTCCAATCAGCCCCAATAAG	*Clostridium botulinum* A2 str. Kyoto (intact prophage)	3	5′-TT**T**T**A**-3′
	AAAAGCCAATCAAAATCTATTTTATATTTAGATTTAAAAGCCAGTCAAAATCTATTAAATATTTAGATTT	*Clostridium botulinum* F str. 230613 (intact prophage)	3	5′-TA**T**A**A**-3′
	AAAGATAAGAGAGAAGGATTACTTCCAGAAGTAGCAAAGACAAGCGAGAAGGGTTGCTTCCAGAAGTCTA	*Bacillus* sp. FJAT-4402 (questionable prophage)	7	5′-CA**T**C**A**-3′
*C. thermocellum* ATCC 27405 (Type I-B)	ATTCGTTTATCTTTATCAAATCACTCCCTCCCTTCAGATTCGTTTGTCTTTATCAAATCACTCCCTCCTTTCAG	*Clostridium stercorarium* subsp. *stercorarium* DSM 8532 (intact prophage)	2	5′-TTT**CA**-3′
	TGATGAAGGACGCTGAAACAGGAATGTTCCAGGCTGTGATGAAGGACGCTGAAACAGGAATGTTTCAGGCCG	*Clostridium clariflavum* DSM 19732 (vicinity of transposase)	2	5′-GGA**CA**-3′
	ACGAAGCAGGTTTATACAGTTTGATATTGAAATCAAACGAATCAGGTTTATACAGTTTAATCTTTTCATCAA	*Staphylococcus* phage vB_SauM_Remus	6	5′-AAT**CA**-3′

^a^Spacer-protospacer mismatches are underlined. In instances where multiple protospacer hits were obtained from a single spacer query, the top hit is provided. Generally, PAM sequences were found to be identical between multiple protospacer hits from a single spacer sequence.

^b^For hits found within bacterial genomes, the location of the protospacer sequence relative to prophage regions and mobile genetic elements is provided in parentheses.

^c^5 nt of adjacent sequence is provided. Potential conserved residues are bolded.

**Table 3:** Summary of clostridial Type I-B CRISPR-Cas loci analyzed to date.

Species	Number of spacers (total)^a^	PAM sequences^b^	PAM^b^	Reference
*C. autoethanogenum* DSM 10061	**22**, **43**, **33** (98)	5′-TAA-3′ 5′-TAA-3′ 5′-CAA-3′ 5′-GAA-3′	5′-NAA-3′	This study^7^
*C. difficile* 630/R20291	1, 2, 1, 1, 4, 2, 4, 3, 2, 14, 11, 4, 5, 4, 14, 9, 26, 9 (116)	**5**′**-CCA-3**′ **5**′**-CCT-3**′	**5**′**-CCW-3**′^c^	62,7
*C. pasteurianum* ATCC 6013	**37, 8** (45)	**5**′**-TCA-3**′ **5**′**-TTG-3**′ **5**′**-TCT-3**′	ND^d^	This study^7^
*C. tetani* 12124569	22, **3, 4, 2, 4, 5, 10, 3** (53)	5′-TAA-3′ 5′-TTA-3′ 5′-TCA-3′	5′-TNA-3′	This study^7^
*C. thermocellum* ATCC 27405	51, 96, **169**, 78, 42 (436)	5′-TCA-3′ 5′-TCA-3′ 5′-ACA-3′	5′-NCA-3′	This study^7^

^a^Spacers corresponding to Type I-B CRISPR-Cas loci analyzed in this study are bolded.

^b^3 nt PAM and PAM sequences are shown. Experimentally-verified motifs are bolded.

^c^W = weak (A or T).

^d^ND = not determined due to highly varied PAM sequences.

**Table 4:** Strains and plasmids employed in this study.

Strain	Relevant characteristics	Source or reference
*Escherichia coli* DH5α	F^-^ *endA1 glnV44 thi-1 recA1 relA1 gyrA96 deoR nupG φ80dlacZΔM15 Δ(lacZYA-argF)U169, hsdR17(r*_*K*_^-^*m*_*K*_^*+*^*), λ*^-^	Lab stock
*Escherichia coli* ER1821	F^-^ *endA1 glnV44 thi-1 relA1? e14*^-^*(mcrA*^-^*) rfbD1? spoT1? Δ(mcrC-mrr)114::IS10*	Lab stock; New England Biolabs
*Clostridium pasteurianum* ATCC 6013	Wild-type	American Type Culture Collection
*Clostridium pasteurianum* ∆*cpaAIR*	Markerless *cpaAIR* deletion mutant	This study
**Plasmid**	**Relevant characteristics**	**Source or reference**
pFnuDIIMKn	M.FnuDII methyltransferase plasmid for methylation of *E. coli*-*C. pasteurianum* shuttle vectors (Km^R^; p15A ori)	49
pMTL83151	*E. coli-Clostridium* shuttle vector (Cm^R^; ColE1 ori; pCB102 ori)	57
pMTL85141	*E. coli-Clostridium* shuttle vector (Cm^R^; ColE1 ori; pIM13 ori)	57
pCas9	*E. coli cas9* and tracrRNA expression vector (Cm^R^; p15A ori)	27
pCas9gRNA-cpaAIR	Type II CRISPR expression vector containing *cas9* and gRNA targeted to the *C. pasteurianum cpaAIR* gene	This study
pCas9gRNA-delcpaAIR	Type II CRISPR genome editing vector derived by inserting a *cpaAIR* deletion editing cassette into pCas9gRNA-cpaAIR	This study
p85Cas9	*cas9* expression vector derived by inserting *cas9* with its native promoter from pCas9 into pMTL85141	This study
p83Cas9	*cas9* expression vector derived by inserting *cas9* and the tracrRNA from pCas9 into pMTL83151	This study
p85delCas9	Derived by deleting the *cas9* promoter from p85cas9	This study
pSpacer18	*C. pasteurianum* protospacer 18 construct lacking flanking sequences	This study
pSpacer18-5′	*C. pasteurianum* protospacer 18 construct including 5′ protospacer-adjacent sequence	This study
pSpacer18-3′	*C. pasteurianum* protospacer 18 construct including 3′ protospacer-adjacent sequence	This study
pSpacer18-flank	*C. pasteurianum* protospacer 18 construct including flanking protospacer-adjacent sequence	This study
pSpacer24	*C. pasteurianum* protospacer 24 construct lacking flanking sequences	This study
pSpacer24-5′	*C. pasteurianum* protospacer 24 construct including 5′ protospacer-adjacent sequence	This study
pSpacer24-3′	*C. pasteurianum* protospacer 24 construct including 3′ protospacer-adjacent sequence	This study
pSpacer24-flank	*C. pasteurianum* protospacer 24 construct including flanking protospacer-adjacent sequence	This study
pSpacer30	*C. pasteurianum* protospacer 30 construct lacking flanking sequences	This study
pSpacer30-5′	*C. pasteurianum* protospacer 30 construct including 5′ protospacer-adjacent sequence	This study
pSpacer30-3′	*C. pasteurianum* protospacer 30 construct including 3′ protospacer-adjacent sequence	This study
pSpacer30-flank	*C. pasteurianum* protospacer 30 construct including flanking protospacer-adjacent sequence	This study
pCParray-cpaAIR	Type I-B CRISPR expression vector containing a synthetic CRISPR array targeted to the *C. pasteurianum cpaAIR* gene	This study
pCParray-delcpaAIR	Type I-B CRISPR genome editing vector derived by inserting a *cpaAIR* deletion editing cassette into pCParray-cpaAIR	This study
