# Supplementary material for: Harnessing heterologous and endogenous CRISPR-Cas machineries for efficient markerless genome editing in Clostridium
Source: Sci Rep. 2016 May 9;6:25666. doi: 10.1038/srep25666 (PMC4860712; doi:10.1038/srep25666)
Supplement: Supplementary Information [file srep25666-s1.pdf]

Harnessing heterologous and endogenous CRISPR-Cas machineries for efficient markerless genome editing in *Clostridium*

Michael E. Pyne[1,†], Mark R. Bruder[1], Murray Moo-Young[1], Duane A. Chung[1,2,3,*], C. Perry Chou[1,*]

Department of Chemical Engineering, University of Waterloo, Waterloo, Ontario, Canada[1]; Department of Pathology and Molecular Medicine, McMaster University, Ontario, Canada[2]; Algaeneers Inc. and Neemo Inc., Hamilton, Ontario, Canada[3];

Running head: Harnessing endogenous CRISPR-Cas loci in *Clostridium*

*Address correspondence to Duane A. Chung, duane.chung@uwaterloo.ca; and C. Perry Chou, cpchou@uwaterloo.ca

†Present address: Department of Biology and Centre for Structural and Functional Genomics, Concordia University, Montréal, Québec, Canada.

Supplementary Figure S1

a)

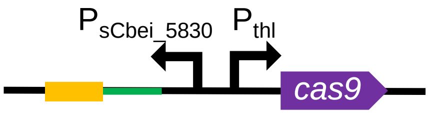

cagtaagcatgacgatccgcggcaaaaaaagcaccgactcggtgccactttttcaagttgataacggactagccttattttaacttgctatttct
agctctaaaacatctgtattagcttcatcagATGGTGGAATGATAAGGGTTTGCACCTTAATTTCTCCTA
TTGAGAAAATCGTCTCTTCTCAGACGTCAAACCATGTTAATCATTGCTTTTATCAAAA
ATATATTTTTAAAATTATTATTAATTTATTAAATGTCATTTTTGATACTTGTTAATGAT
AACACATAAAAAGTCTAAATTCAAGAACATTATTACTGTCTTTATGTTAAATTTATTC
CATACATACCATAATACAATAACAAAATTAAATTTATATCAATTCTTCCCTCATATTT
TTTATTTAAATAAGCCTTAAATCTTTTCAAATTAAAGATTATttacagaagtcgaggagctactGG
TAAAATCTATTGATTAAAAAAATATTTGTGGTTATAATTAAATTGTGAATTAAATAA
CAATCGATTTGTGTATTTATAAGAATATAAACTTTAGGAGGTTACTTTTTatggataagaaat
actcaataggcttagatatcggcacaaatagcgtcggatgggcggtgatcactgatgaatataaggttccgtctaaaaagttcaaggttctgg
gaaatacagaccgccacagtatcaaaaaaaatcttataggggctcttttatttgacagtggagagacagcggaagcgactcgtctcaaacgg
acagctcgtagaaggtatacacgtcggaagaatcgtatttgttatctacagg

b)

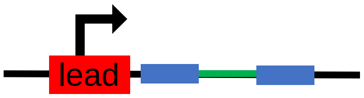

Cattcagagctcggatggttaacagtgctagaaaatagatatatctataatttaatttagtaaattgataatattcaataagattttaccaagtaag
ataaaaataaagatacctatgaagtacttatacataaggcttataggtgtttttctattaaaatttacgtaagactaaaaatagctggtaaatttttg
ctaaatcctttatttttaatgaatagagcattataattatagtaaagaatggctagttttaagtagttgaaccttaacataggatgtatttaaatcaga
aaataagtcttctgtatatactaatcctgattgttgaaccttaacataggatgtatttaaatgttaaaataaaggataaatgattaataaatatgttata
atattaattatctaatatttaaattaaggatgcgattttattacggatagaacagagttattaaatgttattaagaatggagaaaattcatatatagaa
ttcaaagaagaagctataaaagcaaaagatttggcagaagaatttgtagcttttgctaatgccgaaggtggaacggtgctaataggaatagct
gacgatggaggcataaaaggggtaactgatagtaatatagaagagaagattatgaatatttgcaggaagagctcatcagg

Supplementary Figure S1. Sequence and structure of synthetic DNA constructs employed in this study. **(a)** 821 bp synthetic gRNA gene synthesis product targeted to the *C. pasteurianum cpaAIR* locus. The synthetic gRNA containing a 20 nt *cpaAIR* spacer tag (green) and *cas9* binding handle (orange) was expressed from the sCbei_5830 small RNA promoter (P_{sCbei_5830}). A

reverse orientation *C. pasteurianum thl* gene promoter (P_{thl}) and partial *cas9* coding sequence (violet) was included for transcriptional fusion of P_{thl} to the *cas9* gene. Promoter-containing regions are shown in uppercase letters and restriction endonuclease recognition sites utilized for cloning (SacII + BstZ17I) are underlined. **(b)** 667 bp synthetic CRISPR array gene synthesis product targeted to the *C. pasteurianum cpaAIR* locus. The synthetic CRISPR array containing a 37 nt *cpaAIR* spacer (green) flanked by 30 nt direct repeats (blue) was expressed from a putative promoter (not identified) within the CRISPR leader sequence (lead; red). SacI recognition sites utilized for cloning are underlined.

Supplementary Table S1. Oligonucleotides employed in this study

Oligonucleotide	Sequence (5’-3’)[*]
Cas9.SacII.S	GTTTAGCCGCGGGGCAGCGCCTAAATGTAGAA
Cas9.XhoI.AS	TCAGCTCTCGAGCAGTCTTGAAAAGCCCCTGTATTACTGC
delcpaAIR.PvuI.S	CTACTACGATCGGTCCTAAAAGCAGGGTATGAAGTCCATTAG
delcpaAIR.SOE.AS	CTTGAGGTCTAGGACTTCTATCTGGGAATAGAATGTTGTTCGATAGGCATCC
delcpaAIR.SOE.S	GGATGCCTATCGAACAACATTCTATTCCCAGATAGAAGTCCTAGACCTCAAG
delcpaAIR.PvuI.AS	GTCAAGCGATCGGCTTAGCTGGTAAGAAGCAAGGTCTT
-cas9.SacII.S	GACGATCCGCGGGGTTACTTTTTATGGATAAGAAATACTCAATAGGC
Cas9.BstZ17I.AS	CCTGTAGATAACAAATACGATTCTTCCGAC
spacer18.AatII.S	GGTAAAATTTGATTGTCCTCATTGCGATGAAGAAAGACGT
spacer18.SacII.AS	CTTTCTTCATCGCAATGAGGACAATCAAATTTTACCGC
spacer24.AatII.S	GGTTGCAATAGAATGTGATAAAGACCATACTCATATGTGACGT
spacer24.SacII.AS	CACATATGAGTATGGTCTTTATCACATTCTATTGCAACCGC
spacer30.AatII.S	GGATAATATGGATTGAAGAGTGTTCAGAAGTTAAATAGACGT
spacer30.SacII.AS	CTATTTAACTTCTGAACACTCTTCAATCCATATTATCCGC
spacer18-5’.AatII.S	GGTTTCAGTAAAATTTGATTGTCCTCATTGCGATGAAGAAAGACGT
spacer18-5’.SacII.AS	CTTTCTTCATCGCAATGAGGACAATCAAATTTTACTGAAACCGC
spacer18-3’.AatII.S	GGGTAAAATTTGATTGTCCTCATTGCGATGAAGAAATAGAAAGACGT
spacer18-3’.SacII.AS	CTTTCTATTTCTTCATCGCAATGAGGACAATCAAATTTTACCCGC
spacer24-5’.AatII.S	GGAAATTGTTGCAATAGAATGTGATAAAGACCATACTCATATGTGACGT

spacer24-5'.SacII.AS	CACATATGAGTATGGTCTTTATCACATTCTATTGCAACAATTTCCGC
spacer24-3'.AatII.S	GGTTGCAATAGAATGTGATAAAGACCATACTCATATGTTTTTAAGACGT
spacer24-3'.SacII.AS	CTTAAAAACATATGAGTATGGTCTTTATCACATTCTATTGCAACCGC
spacer30-5'.AatII.S	GGTATCTATAATATGGATTGAAGAGTGTTCAGAAGTTAAATAGACGT
spacer30-5'.SacII.AS	CTATTTAACTTCTGAACACTCTTCAATCCATATTATAGATACCGC
spacer30-3'.AatII.S	GGATAATATGGATTGAAGAGTGTTCAGAAGTTAAATATGCTGGACGT
spacer30-3'.SacII.AS	CCAGCATATTTAACTTCTGAACACTCTTCAATCCATATTATCCGC
spacer18-flank.AatII.S	GGTTTCAGTAAAATTTGATTGTCCTCATTGCGATGAAGAAATAGAAAGACGT
spacer18-flank.SacII.AS	CTTTCTATTTCTTCATCGCAATGAGGACAATCAAATTTTACTGAAACCGC
Spacer24-flank.AatII.S	GGAAATTGTTGCAATAGAATGTGATAAAGACCATACTCATATGTTTTTAAGACGT
Spacer24-flank.SacII.AS	CTTAAAAACATATGAGTATGGTCTTTATCACATTCTATTGCAACAATTTCCGC
Spacer30-flank.AatII.S	GGTATCTATAATATGGATTGAAGAGTGTTCAGAAGTTAAATATGCTGGACGT
Spacer30-flank.SacII.AS	CCAGCATATTTAACTTCTGAACACTCTTCAATCCATATTATAGATACCGC
cpaAIR.S	CATAACCTCAGCCATATAGCTTTTACCTACTCC
cpaAIR.AS	ATAGGTGGATTCCCTTGTCAAGATTTTAGC

* Underline: restriction recognition sequences
